# Use of complementary alternative medicine for low back pain consulting in general practice: a cohort study

**DOI:** 10.1186/1472-6882-7-42

**Published:** 2007-12-18

**Authors:** Jean-François Chenot, Annette Becker, Corinna Leonhardt, Stefan Keller, Norbert Donner-Banzhoff, Erika Baum, Michael Pfingsten, Jan Hildebrandt, Heinz-Dieter Basler, Michael M Kochen

**Affiliations:** 1 Dpt. of General Practice, University of Göttingen, Humboldtallee 38, 37073 Göttingen, Germany; 2 Dpt. of General Practice, Preventive and Rehabilitation Medicine, University of Marburg, Robert-Koch-Str. 5, 35033 Marburg, Germany; 3 Institute for Medical Psychology, University of Marburg, Bunsenstr. 3, 35037 Marburg, Germany; 4 Dpt. of Public Health Sciences, University of Hawaii at Manoa, 1960 East-West Rd., Honolulu, HI 96822, USA; 5 Dpt. of Anaesthesiology, Pain Clinic, University of Göttingen, Robert-Koch-Str. 40, 37075 Göttingen, Germany

## Abstract

**Background:**

Although back pain is considered one of the most frequent reasons why patients seek complementary and alternative medical (CAM) therapies little is known on the extent patients are actually using CAM for back pain.

**Methods:**

This is a post hoc analysis of a longitudinal prospective cohort study embedded in a RCT. General practitioners (GPs) recruited consecutively adult patients presenting with LBP. Data on physical function, on subjective mood, and on utilization of health services was collected at the first consultation and at follow-up telephone interviews for a period of twelve months

**Results:**

A total of 691 (51%) respectively 928 (69%) out of 1,342 patients received one form of CAM depending on the definition. Local heat, massage, and spinal manipulation were the forms of CAM most commonly offered. Using CAM was associated with specialist care, chronic LBP and treatment in a rehabilitation facility. Receiving spinal manipulation, acupuncture or TENS was associated with consulting a GP providing these services. Apart from chronicity disease related factors like functional capacity or pain only showed weak or no association with receiving CAM.

**Conclusion:**

The frequent use of CAM for LBP demonstrates that CAM is popular in patients and doctors alike. The observed association with a treatment in a rehabilitation facility or with specialist consultations rather reflects professional preferences of the physicians than a clear medical indication. The observed dependence on providers and provider related services, as well as a significant proportion receiving CAM that did not meet the so far established selection criteria suggests some arbitrary use of CAM.

## Background

Low back pain (LBP) is a major health problem in industrialized countries with significant economic impact [1]. Although it is one of the most common conditions for which adults seek medical attention, there are still few therapeutic interventions with proven clinical benefit [2]. Patients suffering from LBP are frequently dissatisfied with conventional treatment options and turn to complementary and alternative medicine (CAM) [3]. In a telephone survey in the United States 54% of the sample reporting back or neck pain had used one form of CAM compared to 34% who consulted a conventional health care provider [4]. In a Canadian survey 39% of patients with chronic LBP reported use of CAM [5].

CAM is also popular in Germany and 73% of German individuals above 16 years have at least used one form of CAM [6]. According to a widespread definition the term CAM is defined as a group of therapeutic and diagnostic disciplines that usually exist outside the institutions where conventional health care is taught and provided [7]. In Germany, parts of CAM are integrated in conventional medical care. Physicians can qualify and get formal accreditation in different specialties relating to CAM, generally offered in addition to a medical speciality. In 2005, the National Association of Statutory Health Insurance Physicians in Germany reported that 9214 (7.8%) physicians in the ambulatory care sector were accredited in spinal manipulation therapy (SMT), 7544 (6.3%) in naturopathic medicine and 2678 (2.3%) in homeopathy [8]. It is estimated that over 20,000 physicians had training in acupuncture [9]. Additionally, many physicians provide CAM without specific training or formal accreditation [10]. Other health care professionals such as naturopathic healers ["Heilpraktiker"] also offer CAM, but are not very popular for LBP [11]. In Germany, unlike in other countries with non-medical chiropractors, practice of manual therapy including manipulation as well as ordering X-rays or running an imaging facility is restricted to physicians. Access to massage usually requires a referral from a licensed physician. While some CAM services, e.g. SMT or acupuncture is offered within special programs, are covered by the statuary health insurance other CAM services are not.

The purpose of this study was to estimate the extent of CAM use for LBP in Germany and to obtain information about the most commonly used CAM methods. Additionally, we explore which disease-related, socio-demographic and healthcare-related factors are associated with CAM use for LBP.

## Methods

### Study design

This is a post hoc analysis of a longitudinal prospective cohort study embedded within a three armed randomized controlled trial (RCT) with an educational intervention in a primary care setting [12]. The present cohort encompasses all patients enrolled in that trial. The primary goal of the RCT was to asses the impact of guideline-oriented treatment on functional capacity in patients with LBP. A predefined secondary goal of the study was to explore the variation of health care services for LBP. The study was conducted in two centers (Marburg, Göttingen). Ethical approval was obtained from both study sites.

### General practitioners

We contacted 818 general practices in the geographical area of both study centers. Addresses were obtained from local health authorities. From 118 practices who agreed to participate, 2 dropped out after randomization. The GPs were on average 12.7 years in practice (SD ± 6.9), 48 years old (SD ± 6) (national average 50.4 years) and 42% of them were female (national average 36%). A total of 68 (59%) practices were run by a single GP. The basic demographic data of our sample is not meaningfully different from the national average [13]. Of the 116 participating practices, 3 (2%) were accredited in homeopathy, 5 (4%) in SMT, 16 (14%) in naturopathic medicine, and 25 (21%) in acupuncture. Additionally, 22 (19%) practices offer transcutaneous electric nerve stimulation (TENS) and 46 (40%) at least one form of electrotherapy.

### Patients

During the recruitment period practice nurses asked every patient with LBP to participate in the study. All patients were registered to allow an estimation of the number of screened patients. Inclusion criteria were (1) consulting for LBP, (2) age above 18, (3) ability to read and understand German, and (4) written consent.

### Instruments and data collection

**Figure 1:**
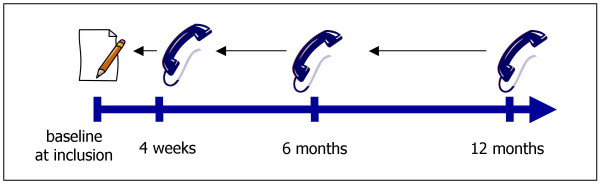
Data collection over 12 months period.

After written consent had been obtained, socio-demographic data were collected with a baseline questionnaire prior to the consultation. During the consultation, GPs assessed warning signs for complicated LBP ("red flags"). Those were major trauma, suspicion or history of cancer, suspicion of inflammatory disease, suspicion of osteoporosis, fever, immunosupression and severe neurological deficits. At follow-ups (four weeks, six months and 12 months later) study nurses conducted standardized telephone interviews and patients were asked about their individual health care utilization, e.g. specialist consultations, medication, and non-pharmacological treatments for LBP within the last 6 months or respectively since inclusion (Figure 1). In the interview, study nurses actively presented a list of 42 possible interventions for LBP. Study nurses were trained in conducting standardized interviews and were able to describe each method in more detail if necessary. We also report some interventions which are not considered as CAM but usually are not recommended as first line therapies by back pain guidelines or are used as household remedies.

The Hanover Functional Ability Questionnaire (HFAQ) was used for the assessment of functional capacity [14]. The HFAQ is a frequently used instrument for the assessment of back pain disability and a scale with good psychometric properties that are comparable to the Roland & Morris Scale [15]. We preferred the HFAQ because it only consists of 12 items without a loss of psychometric quality compared to the Roland & Morris Scale which is advantageous in telephone interviews. The scale ranges from 0 (extreme functional limitation) to 100 (no functional limitation); scores below 70 are considered to represent a significant impairment.

To classify the natural history of LBP, we used a modification of the von Korff procedure as follows [16]:

▪ **Acute LBP**: single episode of LBP of less than 90 days duration

▪ **Recurrent LBP**: multiple episodes LBP of less then 90 days duration within the last 12 months

▪ **Chronic LBP**: more than 90 consecutive days of LBP within the last 12 months.

To estimate the proportion of patients with radicular symptoms, we relied on the patients' reported level of pain radiation into the leg, which we considered as an indicator of possible nerve root irritation. Given the absence of reliable methods, this is a frequently used and pragmatic approach for assessing radicular pain in large cohorts [17].

For the assessment of depression, we applied the German version of the Center for Epidemiologic Studies – Depression Scale (CES-D) [18]. Scores above 23 are considered as possible indicator of a clinically relevant depression [19].

Consultation of orthopedic surgeons, general surgeons and neurologists was summarized as "specialist consultation".

### 2.5. Statistical analysis

We performed logistic regression analyses modeled towards receiving a specific health care service with all socio-demographic and disease-related and healthcare-related factors in Table 1. This procedure provides odds ratios and 95% confidence intervals. Continuous data on depression, pain as measured on a numeric rating scale (NRS) ranging from 0 to 10 and functional capacity were dichotomised. For depression (CESD), we used a cut-off score of >23, for pain levels above 5 and for functional capacity (HFAQ) a cut-off score of >70. All p-values are two-sided and the significance level was 5%.

**Table 1:** Sociodemographic and clinical data.

**Sociodemographic data n = 1342**	**n (%)**
Age groups	
< 40 years	348 (35%)
40–60 years	592 (49%)
> 60 years	263 (22%)
Gender female	778 (58%)
School education	
< 10 years	2859 (21%)
10 years	551 (41%)
> 10 years	506 (38%)
Employment status	
Working full or part-time	765 (57%)
Housekeeping	203 (15%)
Retired	254 (19%)
Unemployed	120 (9%)
Severity of pain > 5 at baseline (scale 1–10)	555 (41%)
Chronicity	
Acute LBP	257 (19%)
Recurrent LBP	536 (40%)
Chronic LBP	550 (41%)
Radiation of pain below the knee*	259 (19%)
(CESD) depression-score > 23 at baseline (n = 1129)	189 (18%)
Suspicion of red flags at baseline	118 (9%)
Functional capacity < 70 at baseline	633 (47%)
Applied for disability pension	97 (7%)
Consulted a specialist within 12 months for LBP	623 (49%)
Rehabilitation	136 (10%)

For acupuncture, SMT, TENS and electrotherapy we added a term in the regression model if such service were provided by the GP.

With a selection procedure (score option) we selected the best model retaining the three most significant predictors. Goodness of fit was tested with the Hosmer-Lemeshow-test and the loglikelihood ratio-test. We report only models in which the null hypothesis of goodness of fit was not rejected [20]. The software package SAS 9.1 was used for analysis.

## Results

### Patients

**Figure 2:**
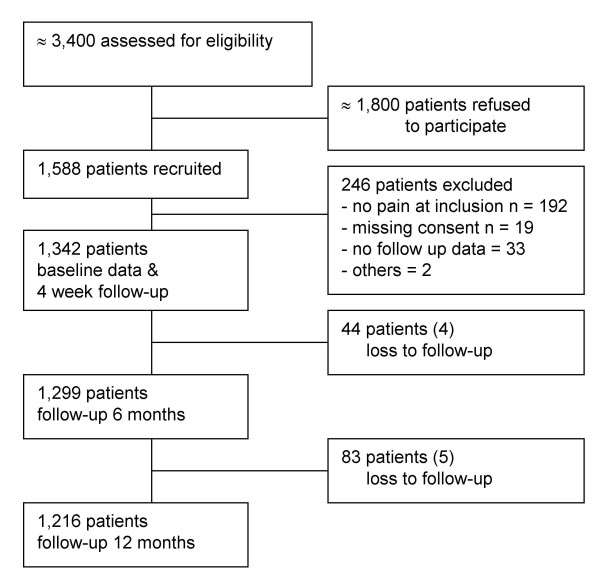
Patient flow.

Over a period of three months the 116 participating practices invited approximately 3,400 patients with LBP to participate in the study. They recruited on average 11.6 (SD ± 5.8) patients; a total of 1,342 of 1,588 patients who agreed to participate were finally included. Patients' flow and reasons for exclusion are listed in Figure 2. During the follow-up period 127 (9.4%) dropped out and 1,218 patients were followed up for one year. Patient characteristics are given in Table 1.

### Utilization of CAM and some other services

In the narrowest to the broadest definitions of CAM service, one half to two thirds of all patients received at least one form of CAM during the one-year follow-up. Using a wide definition including all therapies listed in Tables 2 and 3, a total of 928 patients (69%) received at least one form of CAM. With a narrow definition (acupuncture, SMT and massage), 691 patients (51%) received CAM.

**Table 2:** Predictors for the use of complementary alternative medicine and some other services for low back pain.

**Description of CAM service received**	**Frequency n (%)**	**Predictor variable**	**Odds ratio (95% CI)**	**P value**
Local heat	476 (34%)	Rehabilitation	2.8 (1.2–6.9)	0.002
		Specialist consultation	1.9 (0.8–4.5)	0.16
		Presence of read flags	0.7 (0.2–3.0)	0.63
Massage	417 (31%)	Rehabilitation	3.8 (2.5–5.4)	<0.0001
		Specialist consultation	2.4 (1.9–3.1)	<0.0001
		Chronicity*		
		Recurrent LBP	1.4 (0.9–2.0)	0.42
		Chronic LBP	1.6 (1.1–23)	0.02
Spinal manipulation	352 (26%)	Specialist consultation	5.8 (4.3–7.9)	<0.0001
		GP offering spinal manipulation	5.8 (3.1–10)	<0.0001
		Age group**		
		Age 40–60	0.7 (0.5–1.1)	0.07
		Age > 60	0.3 (0.2–0.5)	0.0009
Electrotherapy	232 (17%)	Rehabilitation	2.4 (1.7–3.7)	<0.0001
		Specialist consultation	1.9 (1.4–2.5)	<0.0001
		Education***		
		10 years	1.1(0.7–1.7)	0.36
		< 10 years	1.6(1.1–2.4)	0.006
Acupuncture	178 (13%)	Specialist consultation	3.8 (1.6–5.8)	<0.0001
		GP offering acupuncture	3.0 (2.1–4.4)	<0.0001
		Chronicity*		
		Recurrent LBP	1.4 (0.8–2.5)	0.63
		Chronic LBP	2.5 (1.4–4.3)	<0.0001
Traction	140 (10%)	Specialist consultation	2.9 (1.9–4.5)	<0.0001
		Rehabilitation	1.6 (1.1–2.7)	0.03
		Chronicity*		
		Recurrent LBP	1.5 (0.8–2.7)	0.79
		Chronic LBP	2.0 (1.1–3.6)	0.01
TENS^§^	121 (9%)	Specialist consultation	3.1 (2.0–4.9)	<0.0001
		Functional capacity < 70	1.9 (1.3–2.9)	0.001
		GP offering TENS^3^	1.7 (1.1–2.6)	0.002
Homoeopathy	41 (3%)	Being female	2.8 (1.3–6.1)	0.009
		Specialist consultation	2.5 (1.2–5.2)	0.012
		Pain on a VAS^§§ ^> 5	2.0 (0.9–4.0)	0.06

* comparison acute LBP

** comparison age below 40

*** comparison > 10 years education

§ Transcutaneus electric nerve stimulation, §§ visual analog scale

**Table 3:** Complementary alternative medicine (CAM) used by less then 40 patients

**CAM**	**n (%)**
Magnet-resonance therapy	28 (≈ 2%)
Underwater pressure massage	18 (< 2%)
Cold therapy	17 (< 2%)
Phytotherapy	8 (< 1%)
Naturopathic healer	22 (< 2%)
Non medical Chiropractor	16 (< 2%)
Osteopath	7 (< 1%)
Cupping	25 (≈ 2%)

In Table 2 the number of patients who received a specific form of CAM and the predictors for receiving this form of CAM are presented. The most popular forms of CAM were local heat, massage and spinal manipulation. The predictors for receiving at least one form of CAM varied, but the consultation of a specialist and a stay in a rehabilitation facility was consistently associated with the use of CAM.

Therapies which were used by less than 40 patients are given in Table 3.

## Discussion

### Summary of main findings

Our study confirms that a large proportion of patients with back pain is at least using one form of CAM, mostly in the form of local heat, massage and spinal manipulation [4,5]. Using CAM was associated with specialist care, chronic LBP and staying in a rehabilitation facility. Receiving spinal manipulation, acupuncture or TENS were associated with consulting a GP who provides these services. Apart from chronicity, disease-related factors like functional capacity or pain only showed weak or no association with receiving CAM.

### Meaning of the results

#### Predictors for the use of CAM and related treatment modalities

Unlike multiple other studies we did not observe the consistently reported association of CAM with younger age, higher educational status and higher income [4,5,21,22]. The lack of association with higher income and higher education might reflect the integration of large parts of CAM like manual therapy, massage and acupuncture into conventional care in Germany, thus requiring no or only small co-payments for CAM services.

We are analysing associated factors for each treatment modality separately instead of treating CAM as a summary variable, which might also partly explain the observed difference.

The consistently observed association between staying in a rehabilitation facility and specialist consultation is an indicator of patients with higher health care demands. This is also reflected by the frequently observed association with chronic LBP, although this was a weaker predictor. But it also reflects traditional treatment strategies and professional preferences. Mood disorders were found to be associated with use CAM by others, but in our sample a positive depression score (CESD) was unrelated [5,21,23]. We did not collect data on comorbid conditions which were also found to be associated with CAM use by others [5,21,23].

#### Local heat and cold

Application of local heat was very popular while only few patients applied cold ant the only significant predictor was stay in rehabilitation. While there is still insufficient evidence regarding the effects of the application of cold for LBP, there is moderate evidence that heat wrap therapy reduces pain and disability for patients with back pain that lasts for less than three months. The relief is relatively small and has only been shown to occur for a short time [24]. Heat wraps are traditionally part of the treatment offered in rehabilitation facilities for patients with chronic LBP, which seems contradictory. But heat wraps are only part of a multimodal approach used to mobilize patients. Less than one third of patients using local heat stayed in rehabilitation. There are several ways to apply heat and we do not know which form was used and if appliance of heat was recommended by health care providers. Appliance of heat is a widespread house hold remedy and some might have used self-applied heat pads that are available over the counter.

#### Massage

Receiving massage for LBP was the most used classical CAM. It is now considered effective for chronic LBP [25,26]. A recent European survey comparing the treatment for chronic pain found a high prevalence for the application of massage therapy in Germany [27]. Our findings confirm this study. Massage is traditionally part of the treatment offered in rehabilitation facilities. Since GPs have to manage a budget for physiotherapy and massage therapy, the purpose of a referral of patients to a specialist might be solely to avoid exceeding their own budget. Specialist might feel pressured to offer an additional treatment going beyond the services already provided by GPs. However, of all patients who received massage within the first four weeks, 13% had acute and 40% recurrent LBP, indicating some arbitrary prescriptions.

#### Spinal manipulation

Not surprisingly, consulting a GP with training in spinal manipulation, acupuncture or TENS was associated with receiving exactly that service. A total of 77% of ambulatory orthopaedic surgeons in Germany have training in SMT, which explains the high amount of spinal manipulation in those cases [6]. Evidence is shifting towards effectiveness of manipulative therapy for acute LBP [25]. This is not reflected by current national and international guidelines based on a Cochrane Review [28]. Most patients receiving manipulative therapy had chronic (47%) or recurrent LBP (39%) and could be considered as inappropriate for manipulation. Older individuals were significantly less likely to receive spinal manipulation which seems reasonable since they are more likely to have contraindications for manipulations, like osteoporosis.

#### Acupuncture

Recent studies found acupuncture to be effective for chronic LBP [29,30]. However, 40% of patients receiving acupuncture hat acute or recurrent LBP. A more detailed report on the use of acupuncture in our sample has been published elsewhere [31]. Acupuncture was apart from provider related factors associated with chronic LBP. It was used as adjuvant therapy and did not result in decreased use of other health care services. A significant proportion (40%) of patients who received acupuncture did not meet the so far only known selection criterion, namely chonicity.

#### Electrotherapy and TENS

The available evidence supporting the use of TENS as isolated treatment modality is limited and conflicting [32]. The association with low functional capacity and specialist consultation indicates that patients receiving TENS were highly affected by LBP.

The term electrotherapy summarizes different forms of electric muscle stimulation, excluding TENS. It is a traditional adjunctive treatment in the management of LBP with proven short term effect on pain [33,34]. Electrotherapy is one of the few instances where a sociodemographic factor (low educational status) was associated. Unlike acupuncture, spinal manipulation and TENS, consulting a GP providing electrotherapy was not associated with receiving electrotherapy. This might reflect a low conviction of GPs that electrotherapy is effective. Patients with lower educational status might also have lower awareness of other forms CAM.

#### Other treatment modalities

Although nearly 10% of the patients, mostly with chronic LBP, received traction therapy there is no conclusive evidence that this treatment has any long term benefit [35].

It has been observed that women are more prone to receive various kinds of CAM [36], but with the exception of homeopathy we found no association between CAM prescription and gender. Only few patients with high level of pain on the NRS used homeopathy for LBP, which is consistent with the experts' opinion that homeopathy is not likely to be effective for LBP [37].

Only a minority of patients obtained treatment from non-physicians like osteopaths, natural healers and non-medical chiropractors. Unlike in other countries these services are not widespread available in Germany because regulations favour physicians to offer this treatments.

#### Strengths and limitations

This is to date the largest prospective cohort study in Germany that provides clinical data and data on the utilization of CAM in a population of primary care patients with LBP. The sample size and the demographic data of the participating GPs make us confident that our observations are representative of current clinical practice in this country. It is possible that we have ignored other important factors that influence the use of CAM like patients' previous experience. Another limitation to the generalizability of our results might be the fact that patients who were more impaired might have been more likely to agree to participate in the study. This might have led to an overestimate of the proportion of patients using CAM. Unfortunately, we do not know how many patients have been offered CAM and how many of them have declined to accept the treatment e.g. due to co-payment or other reasons. Although we believe that only few people in Germany seek care for LBP without consulting a physician. Our results might underestimate the use of CAM in the whole population, particularly for non-prescription herbal medicine. It has been shown that patients seeking care from chiropractors differ from patients seeking care from medical doctors alone [38].

## Conclusion

The frequent use of CAM for LBP shows that CAM is popular in patients and doctors alike. The consistently observed association between prescription of CAM and staying in a rehabilitation centre and specialist consultations is indicating that CAM is mainly used for managing patients with higher and healthcare demands and chronic LBP. These factors are stronger predictors than any back pain related or sociodemographic item we collected as found in many other surveys. However, the observed dependence of CAM use on providers and provider-related services, as well as a significant proportion receiving CAM that did not meet the so far established selection criteria suggest some arbitrary use of CAM.

Evidence for effectiveness of CAM is increasing; therefore the next step in research would be to identifying characteristics of patients with LBP which will most benefit from a specific form of CAM.

## List of abbreviations

**CAM**: complementary alternative medicine, **GP**: general practitioner, **LBP**: low back pain, **NRS**: numeric rating scale, **OR**: odds ratio, **SD**: standard deviation; **SMT**: spinal manipulation therapy, **TENS**: transcutaneus electric nerve stimulation,

## Competing interests

The author(s) declare that they have no competing interests.

## Authors' contributions

All authors contributed to study design. AB, EB, CL, JFC, MP, SK and NDB contributed to data collection. JFC, AB, SK and CL wrote the analysis plan, JFC, AB and CL analysed data. JFC and MMK were the principal authors of the manuscript; JFC had full access to all data, and is the guarantor. All authors contributed to manuscript drafting and revision and approved the final manuscript.

## Pre-publication history

The pre-publication history for this paper can be accessed here:


